# Restoration of dietary-fat induced blood–brain barrier dysfunction by anti-inflammatory lipid-modulating agents

**DOI:** 10.1186/1476-511X-11-117

**Published:** 2012-09-17

**Authors:** Menuka Pallebage-Gamarallage, Virginie Lam, Ryusuke Takechi, Susan Galloway, Karin Clark, John Mamo

**Affiliations:** 1 School of Public Health, Curtin University, Kent Street, Bentley, 6102, Western Australia; 2 Australian Technology Network, Centre for Metabolic Fitness, GPO Box U1987, Perth, 6845, Australia

**Keywords:** Alzheimer’s disease, Blood–brain barrier, Atorvastatin, Pravastatin, Ibuprofen, Saturated-fatty acids

## Abstract

**Background:**

Several studies have identified use of non-steroidal-anti-inflammatory drugs and statins for prevention of dementia, but their efficacy in slowing progression is not well understood. Cerebrovascular disturbances are common pathological feature of Alzheimer’s disease. We previously reported chronic ingestion of saturated fatty acids (SFA) compromises blood–brain barrier (BBB) integrity resulting in cerebral extravasation of plasma proteins and inflammation. However, the SFA-induced parenchymal accumulation of plasma proteins could be prevented by co-administration of some cholesterol lowering agents. Restoration of BBB dysfunction is clinically relevant, so the purpose of this study was to explore lipid-lowering agents could reverse BBB disturbances induced by chronic ingestion of SFA’s.

**Methods:**

Wild-type mice were fed an SFA diet for 12 weeks to induce BBB dysfunction, and then randomised to receive atorvastatin, pravastatin or ibuprofen in combination with the SFA-rich diet for 2 or 8 weeks. Abundance of plasma-derived immunoglobulin-G (IgG) and amyloid-β enriched apolipoprotein (apo)-B lipoproteins within brain parenchyme were quantified utilising immunofluorescence microscopy.

**Results:**

Atorvastatin treatment for 2 and 8 weeks restored BBB integrity, indicated by a substantial reduction of IgG and apo B, particularly within the hippocampus. Pravastatin, a water-soluble statin was less effective than atorvastatin (lipid-soluble). Statin effects were independent of changes in plasma lipid homeostasis. Ibuprofen, a lipid-soluble cyclooxygenase inhibitor attenuated cerebral accumulation of IgG and apo B as effectively as atorvastatin. Our findings are consistent with the drug effects being independent of plasma lipid homeostasis.

**Conclusion:**

Our findings suggest that BBB dysfunction induced by chronic ingestion of SFA is reversible with timely introduction and sustained treatment with agents that suppress inflammation.

## Background

Accumulating evidence is consistent with the concept that the onset and progression of Alzheimer’s disease (AD) is influenced by vascular-risk factors. A number of studies have demonstrated a positive association between AD and atherosclerosis, cardiovascular disease, dyslipidaemia, hypertension and insulin resistance [1,2]. Population studies have also demonstrated that consumption of diets which compromise vascular integrity, such as those enriched in saturated-fatty acids, trans-fatty acids, or cholesterol are also associated with increased risk of AD [3-5]. Moreover, recent animal model and clinical studies suggest that cerebral capillary dysfunction may develop with ageing in the absence of other significant comorbidities [6-8]. Clearly, identifying strategies to prevent or regress this age-induced effect on cerebrovascular function is a therapeutic priority given the aging population of developed and developing countries.

Accumulating evidence suggests that cerebral capillary dysfunction precedes amyloidosis, a hallmark pathological protein marker for Alzheimer’s disease [9]. Common vascular pathological alterations prior to amyloid deposition include a reduction of cerebral capillary endothelial tight junction proteins and increased endothelial pinocytic activity, which in combination result in parenchymal extravasation of plasma proteins within brain parenchyma [10-13]. Activation of glial cells and mitochondrial respiration are markedly increased, altering the phenotypic properties of astrocytes. In response to cytokine production by the latter, parenchymal penetrance of circulating monocytes may subsequently occur [10]. Thereafter, deposition of extracellular proteoglycans and collagen reduce arterial distensibility and may cause gross convolutional abnormalities including total capillary collapse with significant alterations in brain blood perfusion [14,15].

Several lines of evidence are consistent with the hypothesis that suppressing cerebral capillary inflammation may confer benefit to AD onset, or disease progression. Reducing the plasma concentration of cytokines and pro-inflammatory proteins by the regular consumption of foods or vitamin supplements that suppress inflammation is associated with a delay for development of dementia [16-19]. Furthermore, attenuation of cerebral capillary inflammatory processes by inhibition of cyclooxygenase (COX) via the use of non-steroidal anti-inflammatory drugs (NSAIDs) may aid in prevention and treatment of AD. Although beneficial properties of NSAIDs in prevention of AD remain controversial [20], some human epidemiological studies suggest that long-term uses of NSAIDs are protective against AD [21]. The adjusted odds ratios (OR) for AD among NSAID users decreased from 0.98 for less than or equal to one year of use, to 0.76 for greater than five years of use [21]. For users of ibuprofen, the OR decreased substantially from 1.03 to 0.56. In a primary prevention study (ADAPT trial) of naproxen (a non-selective COX inhibitor) and celecoxib (a COX-2 selective inhibitor), a 4-year follow-up assessment revealed that subjects previously exposed to naproxen were protected from the onset of AD by 67% compared to placebo [22]. Further analysis of the study identified reduced AD incidence in asymptomatic individuals. However, there were adverse effects at later stages of AD [23]. Therefore, it could be hypothesised that the chronic use of selected and non-specific NSAIDs may be beneficial in the early stages of AD. Direct evidence of an early preventive effect comes from animal studies, which have shown that a range of both COX-1 and COX-2 inhibitors can reduce plaque burden in AD mice and improve cognition in others [24,25].

Population studies also support a role for anti-inflammatory lipid lowering agents in the prevention of dementia. The 3-City Study represents a cohort of more than 9,000 subjects examining the association of plasma cholesterol and lipid-lowering agent intake with dementia prevalence [26]. In that cohort, 2% of participants were demented at baseline. Of the remainder, 30% of the subjects had been prescribed either HMGCoA reductase inhibitors (statins) or peroxisome proliferator activated receptor agonists (fibrates). The HMGCoA reductase inhibitors are widely used lipid-lowering agents that effectively reduce cardiovascular disease risk by not only a reduction in plasma cholesterol, but additionally pleiotropic anti-inflammatory properties. The study observed the OR for dementia was significantly lower among users of lipid lowering agents (OR = 0.61) compared with subjects taking no lipid lowering agents; the effect was similar between statin and fibrate users. The odds for dementia were increased in subjects with hyperlipidemia that were not treated with statins (OR = 1.43). This particular observational study suggests that anti-inflammatory lipid lowering agents could be associated with decreased risk of dementia. However, following adjusting for multiple co-founders, no association was found between lipid lowering agent intake in late life and reduced risk of dementia [27].

Presently, there is insufficient evidence to recommend statins for the treatment of dementia once disease is established or advanced [28]. However, in a cohort of approximately 3,100 subjects, the adjusted hazards ratio for developing AD was substantially reduced in subjects who commenced statin therapy at an earlier age [29]. This finding may explain paradoxical clinical studies, with only some statin interventions demonstrating benefit in subjects with AD [30,31]. Interestingly, a study in normolipemic spontaneously hypertensive rats supports the notion that statins have beneficial cerebral capillary effects; where atorvastatin was shown to prevent blood–brain barrier (BBB) dysfunction [32]. In culture studies of rat brain endothelial cells, pitavastatin was reported to strengthen the BBB integrity [33].

In a clinical context restoration of cerebral capillary integrity would be therapeutically beneficial for slowing or delaying AD progression, although presently this is a difficult phenomenon to assess *in vivo*. Despite this difficulty, proof-of-concept data using surrogate markers of cerebrovascular inflammation in relevant animal models would provide information on the putative efficacy of selected and targeted interventions. Our preliminary investigations demonstrated beneficial effects of atorvastatin in preventing saturated-fatty-acid (SFA) induced cerebrovascular dysfunction [34]. However, restoration by anti-inflammatory agents of BBB function has not been previously investigated. In this study, following, dietary SFA-induced disturbances of BBB integrity, mice were randomised to receive atorvastatin, pravastatin, or ibuprofen. The brain parenchymal extravasation of large molecular weight plasma proteins, including apolipoprotein (apo)-B lipoproteins that are endogenously enriched in amyloid-β (Aβ) was assessed.

## Results

**Figure 1:**
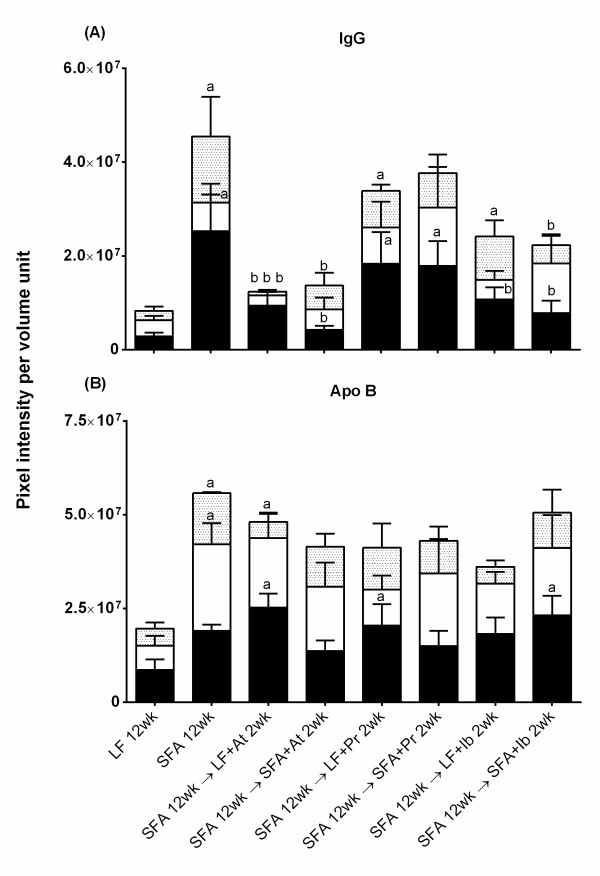
**Quantition of cerebral IgG and apo B in mice randomised to drug diets for 2 weeks.** The bar graphs show 3-dimensional (3D) quantitative analysis of IgG (frame A) and apo B (frame B) leakage of wild-type mouse brains at 2 weeks of drug consumption. Pixel intensity of IgG and apo B was quantitated surrounding the cerebrovasculature in mice fed low-fat (LF), saturated-fatty acids (SFA) and SFA mice randomised to diets containing atorvastatin (LF + At, SFA + At), pravastatin (LF + Pr, SFA + Pr) and ibuprofen (LF + Ib, SFA + Ib). Immunoglobulin-G and apo B pixel intensity was measured in the cortex (CTX, black column), brain-stem (BS, white column) and hippocampal formation (HPF, dotted column) and expressed as per unit volume. Symbol → indicate that mice were fed SFA diet for 12 weeks and then the diet was replaced (→) with a drug containing diet. The bars represent mean intensity and standard error of mean, where *P* < 0.05 considered statistically significant. a: statistically significant in comparison to LF. b: statistically significant in comparison to SFA.

**Figure 2:**
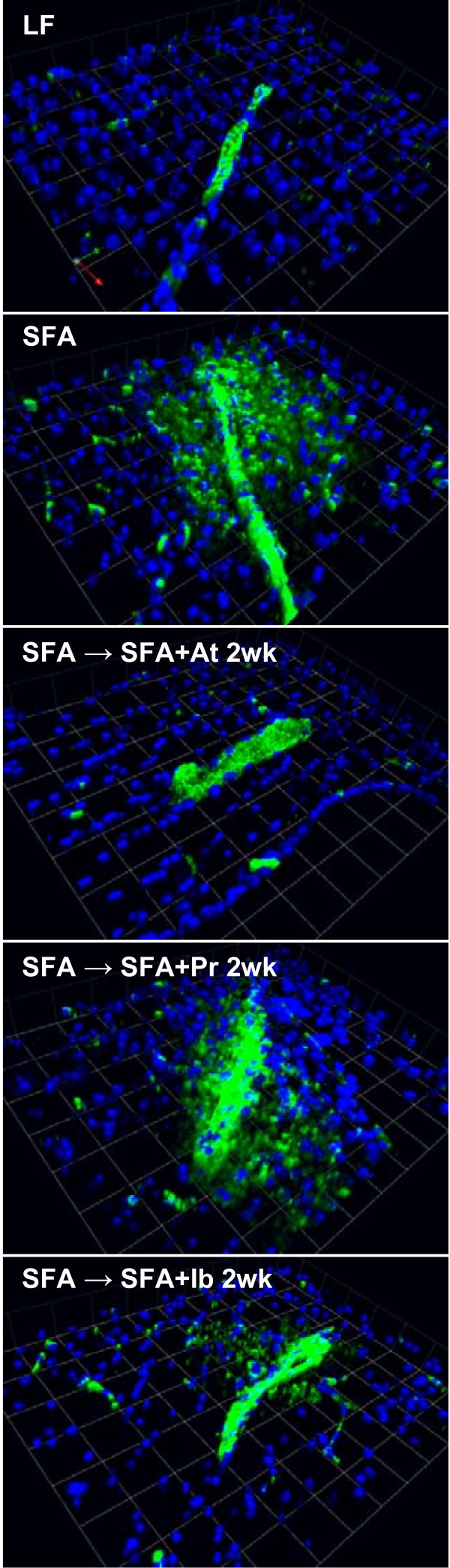
**3-D immunofluorescent staining of cerebral IgG in mice randomised to diet + drug for 2 weeks.** Parenchymal leakage of IgG (green) is observed surrounding the cerebral microvessels. Nuclei are shown in blue. The 3D images are from mice fed low-fat (LF), saturated-fatty acids (SFA) and SFA mice randomised to drug diets containing atorvastatin (LF + At, SFA + At), pravastatin (LF + Pr, SFA + Pr) and ibuprofen (LF + Ib, SFA + Ib) for 2 weeks. Scale: 1 unit = 42.7 *μ*m.

Our results confirm the significant abundance of immunoglobulin-G (IgG) and distribution within the cortex (CTX), brainstem (BS) and hippocampal formation (HPF) in SFA fed wild-type (WT) (Figure 1 and Figure 2). The SFA fed mice had a five-fold greater abundance of IgG compared to the low fat (LF)-control mice, with the majority of this accumulation being indicated within the CTX > HPF > BS. However, HPF had the largest increase in IgG as a consequence of SFA feeding compared to the LF-control fed mice. Following SFA feeding for 12 weeks, provision of atorvastatin, pravastatin or ibuprofen for 2 weeks generally reduced the total parenchymal IgG abundance, however there were differential effects of the agents with respect to efficacy and tissue distribution. The abundance of IgG was essentially completely reversed with atorvastatin, a lipid soluble drug and notably, completely ameliorated the HPF accumulation. In contrast, the IgG distribution in mice given water-soluble pravastatin was not significantly different compared to the SFA treated group. Ibuprofen attenuated IgG as effectively as mice provided with atorvastatin. There was no statistical evidence of an interactive effect of drug with diet regimen (i.e., LF vs SFA) with drug.

**Figure 3:**
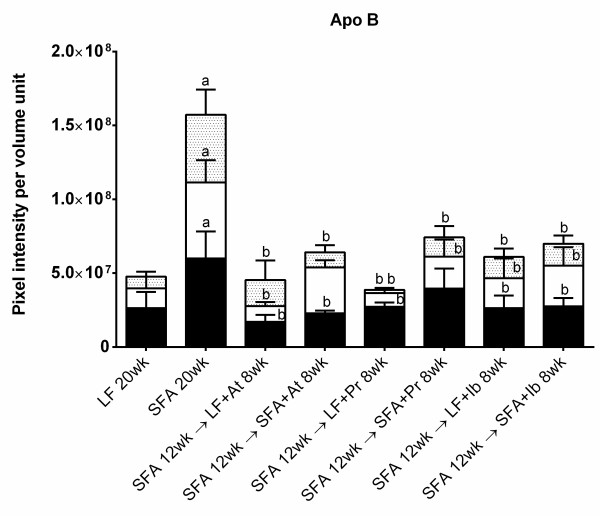
**Quantitation of cerebral apo B in mice randomised to diet + drug for 8 weeks.** The bar graphs show 3-dimensional (3D) quantitative analysis of apo B leakage of wild-type mouse brains. Pixel intensity of apo B was quantitated surrounding the cerebrovasculature in mice fed low-fat (LF), saturated-fatty acids (SFA) and SFA mice randomised to diets containing atorvastatin (LF + At, SFA + At), pravastatin (LF + Pr, SFA + Pr) and ibuprofen (LF + Ib, SFA + Ib). Apo B pixel intensity was measured in the cortex (CTX, black column), brain-stem (BS, white column) and hippocampal formation (HPF, dotted column) and expressed as per unit volume. Symbol → indicate that mice were fed SFA diet for 12 weeks and then the diet was replaced (→) with a drug containing diet.The bars represent mean intensity and standard error of mean, where *P* < 0.05 considered statistically significant. a: statistically significant in comparison to LF**.** b: statistically significant in comparison to SFA.

**Figure 4:**
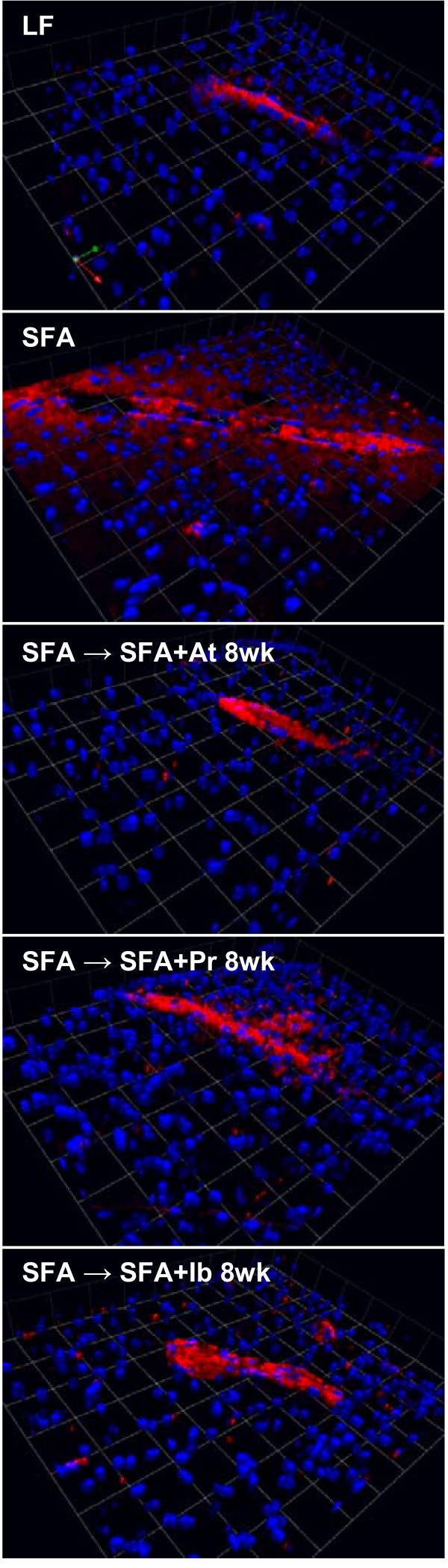
**3-D immunofluorescent staining of cerebral apo B in mice randomised to diet + drug for 8 weeks.** Parenchymal leakage of apo B (red) is observed surrounding the cerebral microvessels. Nuclei are shown in blue. The 3D images are from mice fed low-fat (LF), saturated-fatty acids (SFA) and SFA mice randomised to drug diets containing atorvastatin (LF + At, SFA + At), pravastatin (LF + Pr, SFA + Pr) and ibuprofen (LF + Ib, SFA + Ib) for 8 weeks. Scale: 1 unit = 42.7 *μ*m.

The longer duration of SFA feeding significantly increased the apo B distribution in the parenchyma (compare y-axis, Figure 1 frame B vs. Figure 3), and this occurred primarily within the HPF. The efficacy of a longer period of intervention with atorvastatin, pravastatin or ibuprofen on apo B parenchymal abundance is depicted in Figure 3 and Figure 4. Provision of atorvastatin, pravastatin and also ibuprofen for 8 weeks completely suppressed the SFA-induced effect (Figure 3). This marked improvement with all three agents included normalisation of the HPF accumulation of apo B lipoproteins. There was no evidence of an interactive effect of drugs with diet.

The SFA diet was generally well tolerated consistent with no significant changes in plasma total cholesterol or triglyceride compared to LF controls (Tables 1 and 2). Indeed, the LF control had modestly higher plasma triglyceride compared to other intervention groups. Weight gain was also similar between all treatment groups relative to duration of experimental design (data not shown). Some differential agent effects were observed. Mice randomised to LF with atorvastatin (LF + At) diet for 8 weeks had significantly lower plasma cholesterol, triglyceride and non-esterified fatty acids (NEFA) concentrations (vs. LF control, Table 2), but this was not observed with the provision of atorvastatin with the SFA diet. Ibuprofen and pravastatin had no substantial effects on plasma lipid or NEFA concentrations. Pearson’s correlation analysis found no association between plasma lipid homeostasis and the parenchymal abundance or distribution of IgG or apo B for any of the interventions described (data not shown).

**Table 1:** Plasma lipid profile of SFA mice randomised to SFA diet + drugs for 2 weeks

			**SFA 12wk **					
					**↓**			
	**LF 12wk**	**SFA 12wk**	**LF + At 2wk**	**SFA + At 2wk**	**LF + Pr 2wk**	**SFA + Pr 2wk**	**LF + Ib 2wk**	**SFA + Ib 2wk**
**Cholesterol (mM)**	1.83 ± 0.20	1.54 ± 0.20	1.68 ± 0.11	1.69 ± 0.05	1.64 ± 0.13	1.91 ± 0.13	1.93 ± 0.12	1.59 ± 0.13
**Triglycerides (mM)**	0.74 ± 0.08	0.34 ± 0.04^a^	0.39 ± 0.03^a^	0.34 ± 0.02^a^	0.36 ± 0.01^a^	0.40 ± 0.04^a^	0.42 ± 0.03^a^	0.31 ± 0.02^a^
**NEFA (mEq/L)**	0.85 ± 0.08	0.68 ± 0.04	0.93 ± 0.05	0.82 ± 0.04	1.02 ± 0.05^b^	0.90 ± 0.08	1.05 ± 0.10^b^	0.79 ± 0.05

The table shows the effects of various feeding regimens on plasma lipids. Plasma total cholesterol, triglycerides and non-esterified fatty acids (NEFA) were measured at the end of the feeding regimen. Data represented as mean ± standard error of mean. Means were compared with non-parametric independent t test, where *P* < 0.05 considered statistically significant.

a: statistically significant in comparison to LF;

b: statistically significant in comparison to SFA.

Abbreviations: LF, low-fat; SFA, saturated fatty acids; At, atorvastatin; Pr, pravastatin; Ib, ibuprofen; wk, weeks.

**Table 2:** Plasma lipid profile of SFA mice switched to diets + drug for 8 weeks

			**SFA 12wk**					
					**↓**			
	**LF 20wk**	**SFA 20wk**	**LF + At 8wk**	**SFA + At 8wk**	**LF + Pr 8wk**	**SFA + Pr 8wk**	**LF + Ib 8wk**	**SFA + Ib 8wk**
**Cholesterol (mM)**	1.64 ± 0.14	1.43 ± 0.08	1.17 ± 0.11^a^	1.55 ± 0.14	1.52 ± 0.17	1.37 ± 0.09	1.81 ± 0.26	1.19 ± 0.11^a^
**Triglycerides (mM)**	0.80 ± 0.12	0.55 ± 0.14	0.40 ± 0.04^a^	0.63 ± 0.09	0.89 ± 0.09^b^	1.00 ± 0.17^b^	0.57 ± 0.07	0.80 ± 0.14
**NEFA (mEq/L)**	0.70 ± 0.04	0.61 ± 0.07	0.50 ± 0.07^a^	0.62 ± 0.05	0.62 ± 0.06	0.51 ± 0.04^a^	0.52 ± 0.04^a^	0.52 ± 0.06^a^

The table shows the effects of various feeding regimens on plasma lipids. Plasma total cholesterol, triglycerides and non-esterified fatty acids (NEFA) were measured at the end of the feeding regimen. Data represented as mean ± standard error of mean. Means were compared with non-parametric independent t test, where *P* < 0.05 considered statistically significant.

a: statistically significant in comparison to LF.

b: statistically significant in comparison to SFA.

Abbreviations: LF, low-fat; SFA, saturated fatty acids; At, atorvastatin; Pr, pravastatin; Ib, ibuprofen; wk, weeks.

## Discussion

We confirm that SFA feeding significantly disrupts BBB integrity and function [35], resulting in exaggerated cerebral extravasation of IgG and apo B, but now extend those findings and show an association of severity of dysfunction with duration of SFA feeding. Immunoglobulin-G and apo B lipoproteins are derived from peripheral circulation with molecular weights of approximately 166 kDa and 2.2-20 × 10^6^ kDa, respectively, and are commonly used as a surrogate markers for BBB permeability. The measurement of cerebral abundance of different molecular weight proteins approximates the extent of vascular permeability in cerebral tissue [36]. Wild-type mice fed a SFA diet for 12 weeks had a 5-fold increase in parenchymal abundance of IgG compared to the LF control and about a 2-fold increase in apo B abundance. However, 20 weeks of SFA consumption resulted in >3-fold increase in cerebral apo B abundance when compared to the LF control. Therefore, our findings suggest that the longer the duration of SFA feeding, the greater degree of vascular permeability.

The protective role of statins and NSAIDs on dietary-induced vascular disorders suggests properties that may attenuate cerebral capillary dysfunction [37,38]. The objective of this study was to assess whether dietary SFA-induced cerebral extravasation of IgG and apo B could be reversed by atorvastatin, pravastatin, or ibuprofen in WT mice. A key finding was that atorvastatin completely abolished the accumulation of brain parenchymal IgG and apo B within 8 weeks of treatment and that restoration of function had commenced within just 2 weeks of treatment. This atorvastatin-induced reversal phenomenon occurred independent of whether the mice were maintained on the LF or SFA diet, suggesting potent effects in the continued presence of a potent dietary vascular insult. Similarly, ibuprofen had regressed cerebral abundance of IgG at 2 weeks of intervention, however the magnitude of its effects were not as profound as atorvastatin. A part systemic-mediated phenomenon is supported by the finding that pravastatin, a water soluble statin with poor diffusion properties through the BBB showed efficacy, albeit at 8 weeks of treatment. Atorvastatin, being lipophilic, can passively diffuse through the BBB allowing more rapid and widespread tissue distribution [39]. Pravastatin requires a rate limiting active transport system for cerebral delivery to occur [39].

Evidence that the beneficial effects of atorvastatin and pravastatin on BBB integrity and function were mediated via regulation of inflammation and not lipid metabolism are suggested by the findings with ibuprofen, a non-selective COX inhibitor. Parenchymal abundance of IgG was substantially reduced following 2 weeks of treatment in mice that had been fed an SFA-enriched diet for 12 weeks. Apo B abundance was also significantly reduced with ibuprofen therapy after 8 weeks, analogous to the findings with atorvastatin.

Regional differences in statin or ibuprofen-induced restoration were observed. Atorvastatin was most effective in normalising hippocampal IgG load at 2 weeks and apo B at 8 weeks of intervention, with only partial restoration in CTX and BS with longer-term (8 week) treatment. Treatment with pravastatin or ibuprofen, although less potent than atorvastatin, nonetheless resulted in a uniform reduction of IgG and apo B abundance within HPF, CTX and BS. Ibuprofen showed evidence of efficacy by 2 weeks in HPF and CTX, but pravastatin had no significant effect within these regions at 2 weeks. Collectively, these findings indicate that atorvastatin was most effective in regression of hippocampal plasma protein abundance independent of duration of intervention. Longer-term intake of pravastatin and ibuprofen was required to ameliorate accumulation of plasma proteins within the HPF.

The HPF is the vital brain region involved in learning, memory and some suggest, particularly vulnerable to oxidative damage induced by inflammation [40,41]. Pathological changes reported in human brain specimens indicate that capillary and larger vessel disturbances occur within the HPF and precede the structural abnormalities that then develop within the temporal cortex and other regions of the brain [42-44]. The findings of previous studies demonstrating the potent effects of an SFA diet on BBB function principally within the HPF and of this study, showing the restorative effects of atorvastatin are therefore potentially of clinical relevance.

The mechanisms by which atorvastatin, pravastatin and ibuprofen influenced BBB integrity were not specifically explored in this study. However, the data show that the improvements in BBB function were not associated with plasma lipid homeostasis. These findings suggest that the diets were well tolerated in our mouse model. Consistent with our previous observations where SFA induced BBB dysfunction was independent of hypercholesterolemia [35]. Many studies have demonstrated pleiotropic effects of statins mediated principally via anti-inflammatory, or suppression of oxidative stress pathways, including endothelial specific protection [32,45,46]. In the context of these findings, the statins were comparable, or in some instances more effective than the commonly utilised COX inhibitor, ibuprofen. Similar findings on BBB protection in other models were reported for atorvastatin in a hypertensive rat model [32]. They demonstrated that disturbance in BBB permeability improvement with atorvastatin were associated with abundance of cerebrovascular tight junction proteins; zonula occludens and occludin; plasma nitric oxide concentration and anti-oxidant homeostasis. Similarly, pravastatin was shown to reduce micro-vascular permeability and restore vascular endothelial function via modulation of endothelial nitric oxide synthase level in micro-vessels of rat mesentery [47].

We previously demonstrated that SFA induced BBB leakage was associated with significant reduction in the tight junction protein occludin [35]. Furthermore, studies have demonstrated increased oxidative stress [48-51] and inflammatory cytokine [52,53] associated vascular endothelial dysfunction in rodents maintained on SFA enriched diets. In the model used in this study, statins and ibuprofen probably enhanced the expression of tight junction proteins, suggesting structural stabilisation and repair of this pivotal capillary network.

A paradoxical finding was that abundance of apo B lipoproteins within brain parenchyme required a longer duration of treatment with statins or ibuprofen to be reduced compared IgG, yet apo B lipoproteins have a molecular weight of up to 100 times greater than IgG. Notionally, penetrance into brain parenchyme of macromolecules such as apo B lipoproteins would become evident more quickly than large proteins such as IgG. However, previous findings have shown significant retention of apo B lipoproteins associated with extracellular matrices including heparin-sulphate proteoglycans, biglycan and decorin [54]. The exaggerated presence of apo B lipoproteins compared to IgG in SFA mice treated with statins or ibuprofen, may be indicative of a relatively slow turnover through brain parenchyma, compared to proteins that more readily diffuse into cerebrospinal fluid, such as IgG. Interestingly, apo B lipoproteins that are enriched with amyloid-beta as a consequence of chronic SFA ingestion show remarkable colocalisation with amyloid-plaque in rodent models of AD [35].

## Conclusion

Dysfunction of the BBB is increasingly recognised in neurodegenerative diseases with cerebral capillary disturbances including AD [55]. Although statin and NSAID use have demonstrated benefits for the prevention of AD, appropriate treatment following disease development is understudied and controversial. The majority of studies focus on pathological accumulation of Aβ within the brain parenchyma and neuronal death.

Evidence showing cerebral capillary dysfunction preceding amyloid deposition is growing. This study provides novel insight into the effects of atorvastatin, pravastatin and ibuprofen on regression and prevention of SFA-induced BBB permeability and preceding amyloidosis. Further studies are required to ascertain the underlying mechanisms of how statins and ibuprofen modulate BBB integrity.

## Methods

### Animals and diet conditions

The Curtin University Animal Experimentation and Ethics Committee approved housing, handling and experimental procedures described for this study. Six-week-old female WT mice (C57BL/6 J) were housed in groups and randomised into the diet or drug treatment groups (6 mice per group). All mice were maintained in a 12 h light and dark cycle room, at 22°C and with free access to water and food. Mice were weighed weekly and average daily diet consumption was recorded.

The LF control group of mice were fed a semi-purified diet (AIN93M, Glen Forrest Stockfeeders, Glen Forrest, Western Australia) containing 4% (w/w) total fat (derived from canola oil) and <1% of total digestible energy from lipids. As previously demonstrated, mice were fed SFA diet containing 20% (w/w) cocoa butter (SF07-050, Glen Forrest Stockfeeders) to induce BBB damage [35]. The SFA diet contained palmitic (16:0) and stearic (18:0) acids as the primary saturated fats (13% w/w). Digestible energy for LF and SFA diets were 15.1 MJ/kg and 18.8 MJ/kg, respectively (Table 3).

**Table 3:** Dietary composition

	**SFA diet**	**LF diet**
Calculated nutritional parameters (%)		
Protein	13.6	13.6
Total Fat	20.3	4
Crude Fibre	4.7	4.7
Acid Detergent Fibre	4.7	4.7
Total Carbohydrate	50	64.9
Digestible Energy	18.8 MJ/kg	15.1 MJ/kg
% Digestible Energy from Lipids	40	n/a
% Digestible Energy from Protein	15	n/a
Calculated fat composition (%)		
Myristic Acid 14:0	0.05	Trace
Pentadecanoic Acid 15:0	0.01	n/a
Palmitic Acid 16:0	5.16	0.2
Megaric Acid 17:0	0.05	n/a
Stearic Acid 18:0	7.31	0.1
Arachidic Acid 20:0	0.24	n/a
Behenic Acid 22:0	0.04	n/a
Tetracosanoic Acid 24:0	0.03	n/a
Palmitoleic Acid 16:1	0.05	Trace
Heptadecenoic Acid 17:1	0.01	n/a
Oleic Acid 18:1 n9	6.62	2.4
Gadoleic Acid 20:1	0.01	n/a
Lenoleic Acid 18:2 n6	0.67	0.8
a Linolenic Acid 18:3 n3	0.05	0.4
g Linolenic Acid 18:3 n6	Not detected	n/a
Arachadonic Acid 20:4 n6	Not detected	Trace
EPA 20:5 n3	Not detected	Trace
DHA 22:6 n3	Not detected	Trace

The table shows the total fatty acid composition of SFA and LF. Vitamin and mineral content were balanced in all diets. The agents were incorporated into either LF or SFA chow at a concentration of 20 mg/kg (w/w) atorvastatin, 23.4 mg/kg (w/w) pravastatin and 333.3 mg/kg (w/w) ibuprofen.

To determine the putative restorative effects of atorvastatin (At), pravastatin (Pr) and ibuprofen (Ib) on BBB damage, WT mice were initially fed with SFA diet for a period of 12 weeks to induce damage to the BBB. The SFA fed mice were then switched to SFA diets containing atorvastatin (SFA **→** SFA + At), pravastatin (SFA **→** SFA + Pr) or ibuprofen (SFA **→** SFA + Ib) to determine the effect of drugs with insult (SFA diet). Mice switched to LF diets containing identical doses of atorvastatin (SFA **→** LF + At), pravastatin (SFA **→** LF + Pr) or ibuprofen (SFA **→** LF + Ib) to determine the effects of drugs in the absence of dietary insult. Animals that were initially fed SFA diet for 12 weeks were switched to the drug containing diets (LF + drug or SFA + drug). They were then sacrificed at two time points, at 2 weeks and 8 weeks after drug intervention, to observe any progressive effects of the drugs on BBB restoration. Mice given LF or SFA alone were run parallel with all experiments and sacrificed at each end point.

The agents were incorporated into either LF or SFA chow at a concentration of 20 mg/kg (w/w) atorvastatin (Lipitor, Pfizer, Australia), 23.4 mg/kg (w/w) pravastatin sodium (Lipostat®, Australia) and 333.3 mg/kg (w/w) ibuprofen (I110, Sigma-Aldrich, New South Wales, Australia). Based on measured consumption rates, the daily ingested dose for each agent approximated three times the highest recommended dose for human studies per unit body. However, the bioavailability of atorvastatin and pravastatin has been reported to be reduced when consumed with food [56,57].

### Tissue collection and sample preparation

Mice were maintained on the indicated diets and weighed weekly. Tissue samples were collected as previously described by Takechi et al. [35]. Mice were anesthetised with pentobarbitone (45 mg/kg i.p.) and were exsanguinated by cardiac puncture. Blood was collected into K-2 EDTA tubes and stored on ice. Plasma was separated by short time, high speed centrifugation at 4°C and stored at −80°C.

Brain tissues were carefully isolated, washed with chilled phosphate buffered saline (PBS, pH 7.4), and the right hemispheres were separated and fixed in 4% paraformaldehyde for 24 h. The tissues were then cryoprotected with 20% sucrose solution at 4°C for 72 h, frozen in isopentane with dry ice and stored at −80°C. For histology and fluorescence microscopy, serial cryo-sections of 18 μm were cut from the right cerebral hemispheres for each mouse and mounted on Polysine slides [35].

### Immunoglobulin-G and apolipoprotein B immunofluorescence

Cerebrovascular leakage of IgG and apo B were detected as previously described [35]. Brain cryosections (18 μm) were air-dried for 30 min, rehydrated with PBS and incubated in blocking serum (10% goat serum) prior to application of the antibodies.

For IgG staining, tissues were incubated with goat anti-mouse IgG-Alexa 488 flurochrome conjugated antibody (Invitrogen) at 1:100 dilution, overnight at 4°C. The sections were then washed with PBS and nuclei were counterstained with DAPI (1:1000) for 5 min at room temperature. Thereafter, the stained sections were mounted with anti-fade mounting medium.

Cerebral apo B was detected by overnight incubation with polyclonal rabbit anti-apo B as the primary antibody (ab20737, Abcam, Cambridge, UK) at 1:500 dilution, at 4°C. Post-overnight incubation, primary antibody was labelled at room temperature with the secondary goat anti-rabbit IgG-Alexa 488 conjugate (Invitrogen) for 2 h. The tissues were then counterstained with DAPI and mounted as per IgG staining method [35].

### Immunofluorescent imaging of and quantitative analysis of cerebral IgG and apo B

Digital images for photomicroscopy were acquired through AxioCam HRm camera (Zeiss Germany) with an AxioVert 200 M inverted microscope by Zeiss (Germany) at × 200 magnification (Plan Neofluar x20 objective, 1.3 numerical aperture). Three-dimensional (3-D) images were captured through ApoTome optical sectioning methodology (Carl Zeiss) [35,58]. Each 3-D image consisted of 6–10 two-dimensional images and the distance between Z-stack slices was 1·225 μm optimised by Nyquist. A minimum of nine 3-D images were randomly captured per mouse, which include 5 images within the CTX and 2 images each from BS and HPF.

Cerebrovascular leakage of plasma proteins IgG and apo B were quantified within the CTX, BS and HPF. The pixel intensity of protein of interest surrounding the blood vessels for each 3-D image was quantitated utilising the automated optical intensity measurement tool in Volocity (Software version 5.5, Perkin Elmer, Melbourne, Australia) and expressed as per unit volume. The investigator was blinded during image capturing and quantitative analysis.

### Plasma cholesterol, triglyceride and NEFA

Plasma Cholesterol and triglycerides were determined in duplicate by enzymatic assays (Randox Laboratories LTD, UK). Non-esterified fatty acids were determined with NEFA-C (ASC-ACOD method, Wako Pure Chemical Industries, Osaka, Japan).

### Statistical analysis

This study utilised 6 mice per group and minimum of nine 3-D images were captured per mouse for detection of IgG and apo B leakage within the CTX, BS and HPF. In each group, 324–540 two-dimensional images were generated for adequate statistical comparison.

Normally distributed data were analysed by parametric one-way analysis of variance to assess the main effects of the dietary SFA, atorvastatin, pravastatin and ibuprofen treatment. The Kruskall-Wallis test was utilised if data was not-normally distributed. Post-hoc comparison of means was done if the associated main effect or interaction was statistically significant within the analysis of variance procedure. *P*-values < 0.05 were considered to be statistically significant.

## Abbreviations

(AD): Alzheimer’s disease; (Aβ): Amyloid-β; (apo B): Apolipoprotein B; (At): Atorvastatin; (BBB): Blood–brain barrier; (BS): Brainstem; (CTX): Cortex; (COX): Cyclooxygenase; (HPF): Hippocampal formation; (Ib): Ibuprofen; (IgG): Immunoglobulin; (LF): Low fat; (SFA): Saturated fatty acid; (NEFA): Non-esterified fatty acids; (NSAIDs): Non-steroidal anti-inflammatory drugs; (OR): Odds ratio; (PBS): Phosphate buffered saline; (Pr): Pravastatin; (WT): Wild-type.

## Competing interests

The authors declare that they have no competing interests.

## Authors’ contributions

MPG carried out the design of project, data collection, immunofluorescence, statistical analysis and drafting of the manuscript. VL and RT assisted in tissue collection and interpretation of data. SG helped in the collection of tissues. KC assisted in critically analysing and drafting of the manuscript. JM conceived the study, helped in data interpretation, drafting of the manuscript, obtaining funding and general supervision of the research group. All authors have approved manuscript for submission.
